# Investigating the genetic makeup of the major histocompatibility complex (MHC) in the United Arab Emirates population through next-generation sequencing

**DOI:** 10.1038/s41598-024-53986-1

**Published:** 2024-02-09

**Authors:** Nour al dain Marzouka, Halima Alnaqbi, Amira Al-Aamri, Guan Tay, Habiba Alsafar

**Affiliations:** 1 Center for Biotechnology, Khalifa University of Science and Technology, Abu Dhabi, United Arab Emirates; 2 Division of Psychiatry, Faculty of Health and Medical Sciences, Medical School, The University of Western Australia, Crawley, WA Australia; 3 School of Medical and Health Sciences, Edith Cowan University, Joondalup, WA Australia; 4 College of Medicine and Health Sciences, Khalifa University of Science and Technology, Abu Dhabi, United Arab Emirates; 5 Department of Biomedical Engineering, Khalifa University of Science and Technology, Abu Dhabi, United Arab Emirates

**Keywords:** Major histocompatibility complex (MHC), Human leukocyte antigen (HLA), United Arab Emirates (UAE), Next generation sequencing (NGS), High-throughput screening, Immunogenetics, Genetics, Immunology

## Abstract

The Human leukocyte antigen (HLA) molecules are central to immune response and have associations with the phenotypes of various diseases and induced drug toxicity. Further, the role of HLA molecules in presenting antigens significantly affects the transplantation outcome. The objective of this study was to examine the extent of the diversity of HLA alleles in the population of the United Arab Emirates (UAE) using Next-Generation Sequencing methodologies and encompassing a larger cohort of individuals. A cohort of 570 unrelated healthy citizens of the UAE volunteered to provide samples for Whole Genome Sequencing and Whole Exome Sequencing. The definition of the HLA alleles was achieved through the application of the bioinformatics tools, HLA-LA and xHLA. Subsequently, the findings from this study were compared with other local and international datasets. A broad range of HLA alleles in the UAE population, of which some were previously unreported, was identified. A comparison with other populations confirmed the current population’s unique intertwined genetic heritage while highlighting similarities with populations from the Middle East region. Some disease-associated HLA alleles were detected at a frequency of > 5%, such as HLA-B*51:01, HLA-DRB1*03:01, HLA-DRB1*15:01, and HLA-DQB1*02:01. The increase in allele homozygosity, especially for HLA class I genes, was identified in samples with a higher level of genome-wide homozygosity. This highlights a possible effect of consanguinity on the HLA homozygosity. The HLA allele distribution in the UAE population showcases a unique profile, underscoring the need for tailored databases for traditional activities such as unrelated transplant matching and for newer initiatives in precision medicine based on specific populations. This research is part of a concerted effort to improve the knowledge base, particularly in the fields of transplant medicine and investigating disease associations as well as in understanding human migration patterns within the Arabian Peninsula and surrounding regions.

## Introduction

The Major Histocompatibility Complex (MHC), located on the short arm of chromosome 6, has attracted immense attention due to the wide range of reported disease associations uncovered by genome-wide association studies (GWAS). Encompassing a genomic region that spans approximately 5 megabases, the MHC contains a repertoire of over 300 highly polymorphic genes that contribute to the complex functional landscape of the MHC, playing important roles in immune response modulation, antigen presentation, and other vital immunological processes^1^. Most of the genetic heritability of the MHC is explained by studies involving the typing of Human Leukocyte Antigen (HLA). The molecules encoded by the HLA genes are cell surface proteins that facilitate antigen presentation and trigger the T-cell component of adaptive immunity, subsequently activating the B-cell arm and the generation of pathogen-specific antibodies^2^.

Most of the haplotype (i.e. HLA alleles located on the same chromosome) information on the MHC required for successful selection of donors for solid organ and hematopoietic stem cell transplantation is provided by genotyping major HLA class I (HLA-A, HLA-C, and HLA-B) and class II (HLA-DRB1, HLA-DQB1, and HLA-DPB1) genes. Mismatched combinations of alleles at these HLA loci (i.e. haplotypes) between the donor and the recipient could lead to life-threatening immunological complications such as graft-versus-host disease (GvHD) or rejection^3^. This implies that when matching for MHC haplotypes, there is an enhanced likelihood of improved survival post-transplantation^4^.

The hallmark of the MHC is characterized by high long-range and uneven population-specific complex linkage disequilibrium (LD), which presents a challenge when selecting underlying causal variants in fine-mapping studies^5^. For instance, due to their distant location, or recombination hotspots, certain allele pairs, like HLA-B and HLA-C, are closely associated with chromosome 6^6^, while other gene pairs exhibit, notably HLA-A display weaker linkage with HLA-B and HLA-C or lack significant linkage altogether^7^.

Many groups have directed their efforts toward cataloging the gene content in the MHC and variations of these^8–10^ and to catalog common, intermediate and well-documented (CIWD) alleles in world populations^11^. It became clear that comprehension of the genetic makeup within a population holds significant importance, especially within populations characterized by high levels of diversity and genetic admixture, particularly in situations where there are small effect sizes caused by relatively rare variants^12^. The complex interaction between demographic events and selective mechanisms, including natural selection, contributes to the shaping of the genetic structure of the MHC. As a result, the MHC region has emerged as an intriguing and valuable region for evaluating genetic diversity across global human populations^13,14^.

For example, the MHC region contains specific combinations of alleles of HLA and non-HLA genes that make up a finite number of population-specific conserved sequences that stretch at a distance of nearly 3 Mb of DNA known as Conserved Extended Haplotypes (CEHs)^15^, or Ancestral Haplotypes (AH)^16^. Those CEHs have been identified in different human populations, including people of European^17^, African^18^, Arabian descent^19^, and Asian descent^20^. More recently, a distribution map summarizing the main global population relations using publicly available HLA frequency data revealed the major gap in the representation of some genetically complex populations, including those of Arabian descent^13^.

An appreciation of the genetic architecture of the MHC of a population is important prior to conducting disease association studies in that specific group. Patterns of genetic variation including the composition of the haplotype within a population can be markers of disease risk associations, yet the majority of genetic disease association studies have been predominantly based on populations of European ancestry^21,22^. The knowledge gap in other racial groups is slowly changing. In populations of the Arabian Peninsula, with notable efforts in populations from Bahrain^23^, Kuwait^24^, and Saudi Arabia^25^, the allelic repertoire is described. The populations of the Arabian Peninsula represent a genetically diverse group, despite their common language, history, and culture. The United Arab Emirates (UAE) is located in the southeast portion of the Arabian Peninsula. It has a population that is an ethnically diverse region shaped by significant bidirectional migrations of people between the African, European, and Asian continents^26^. The early inhabitants of the Arabian Peninsula led a nomadic lifestyle, traveling throughout the peninsula in search of waterholes and establishing communities that were centers for trade and cultural exchange. Trade routes increased gene flow into and out of Arabia, resulting in the current diversity of modern Arabia. Building upon the global endeavor of improving the comprehensiveness of the HLA global variation map^13^, this study examined the MHC landscape of the UAE population using alleles inferred from next-generation sequencing (NGS) data.

Previously, the UAE was represented in a dataset of 200 samples that were typed by using the sequence-specific primer (SSP) method^27^. In another study, a cohort of 115 UAE nationals was used to screen the HLA genes with a focus on COVID-19 severity in the UAE population using targeted sequencing^28^. Another cohort of 52 blood donors from Abu Dhabi was analyzed using the SSP method and was made publicly available^29^, (www.allelefrequencies.net). A number of studies have examined families of the UAE population^27,30^, had low-resolution HLA typing as in Kulski et al.^31^ with a cohort of 95 samples, or focused on novel alleles in a single individual as in Abdrabou et al.^32^. In this study, we investigate the MHC landscape of the UAE population within a more extensive cohort than those previously published. We used HLA alleles inferred from next-generation sequencing, specifically obtained through whole genome sequencing and whole exome sequencing.

## Materials and methods

### Ethical declaration

All participants provided written informed consent and completed a questionnaire authorized by the Institutional Review Board (IRB) committee of Mafraq Hospital (MAF-REC 07/2016 04) and the Dubai Health Authority (DSREC-07-2020_39 and DSREC-07/2020_19). For participants under 18 years of age, parental written consent was obtained at the time of sample collection. All procedures were conducted in accordance with the appropriate guidelines and regulations authorized by the IRB committee at Mafraq Hospital and the Dubai Health Authority. Furthermore, all samples were de-identified before their use in the study.

### Sample collection

A total of 570 unrelated, healthy individuals from various regions of the UAE, including the northern, western, eastern, and south-eastern areas, were recruited for the study. Blood samples were collected using a 2 ml sterilized tube with ethylenediaminetetraacetic acid (EDTA). The samples were then transported in a sealed biohazard bag using a cool transport container to the laboratory for genotypic testing. All the participants were UAE citizens. However, no information on sub-ethnicity or country of ancestry was collected. To limit any HLA-related disease association bias, subjects who reported an autoimmune condition (e.g., T1D) were excluded from the study.

### Whole genome sequencing (WGS)

Whole genome libraries were prepared using the protocol recommended by the Illumina TruSeq® DNA PCR-Free Library Prep kit (Illumina Inc., San Diego CA, USA). After quality control using The Kapa Library Quantification Kit for Illumina platforms (ROX low qPCR mix) (Kapa Biosystems, Wilmington MA, USA) and Advanced Analytical Fragment Analyzer (Advanced Analytical Technologies Inc., Ankeny IA, USA), the indexed paired-end NGS library was then loaded into NovaSeq 6000 (Illumina Inc., San Diego CA, USA) for paired-end sequencing.

### Whole exome sequencing (WES)

Whole exome libraries were prepared using the recommended protocol by the Illumina TruSeq Exome Library Prep kit (Illumina Inc., San Diego, CA, USA). The indexed paired-end libraries were then quantified using the Denovix DS-11 FX Fluorometer and the fragment size was determined using the Advances Analytical Fragment Analyzer (Ankeny, IA, USA) for optimum loading into NextSeq 500 (Illumina Inc., San Diego, CA, USA).

### High-resolution HLA typing by targeted sequencing

Thirty-six samples were selected for high-resolution HLA typing to validate the HLA type inference results using the Holotype HLA 96/11 library kit (Omixon, Budapest, Hungary). The HLA types obtained were subsequently used as a gold standard for evaluating the performance of the HLA calling tools using the methods outlined below. We refer to this dataset as gold-standard hereafter.

### Next-generation sequencing data preprocessing for HLA calling

The HLA allele calling was performed for 142 WGS samples, including 119 UAE WGS samples from Daw Elbait et al.^33^ and 428 UAE WES samples. The bioinformatics tools xHLA^34^ and HLA-LA^35^ were used for allele calling due to their well-documented performance in NGS data, particularly in WGS contexts^35^. Only the first two fields of the HLA alleles (i.e. high-resolution typing) were considered in this study. The 'two fields' refers to the first two sets of digits in the HLA allele nomenclature^36^. For the xHLA and HLA-LA downstream analysis, the relevant reads from the HLA region on chromosome 6, chromosome 6 alternative contigs, and HLA contigs were extracted. Default settings were used for both tools. The unmapped reads were ignored to reduce the analysis time without affecting the results since all HLA-related reads had already been mapped within the previous region and contigs.

### Performance evaluation of HLA calling tools

**Figure 1:**
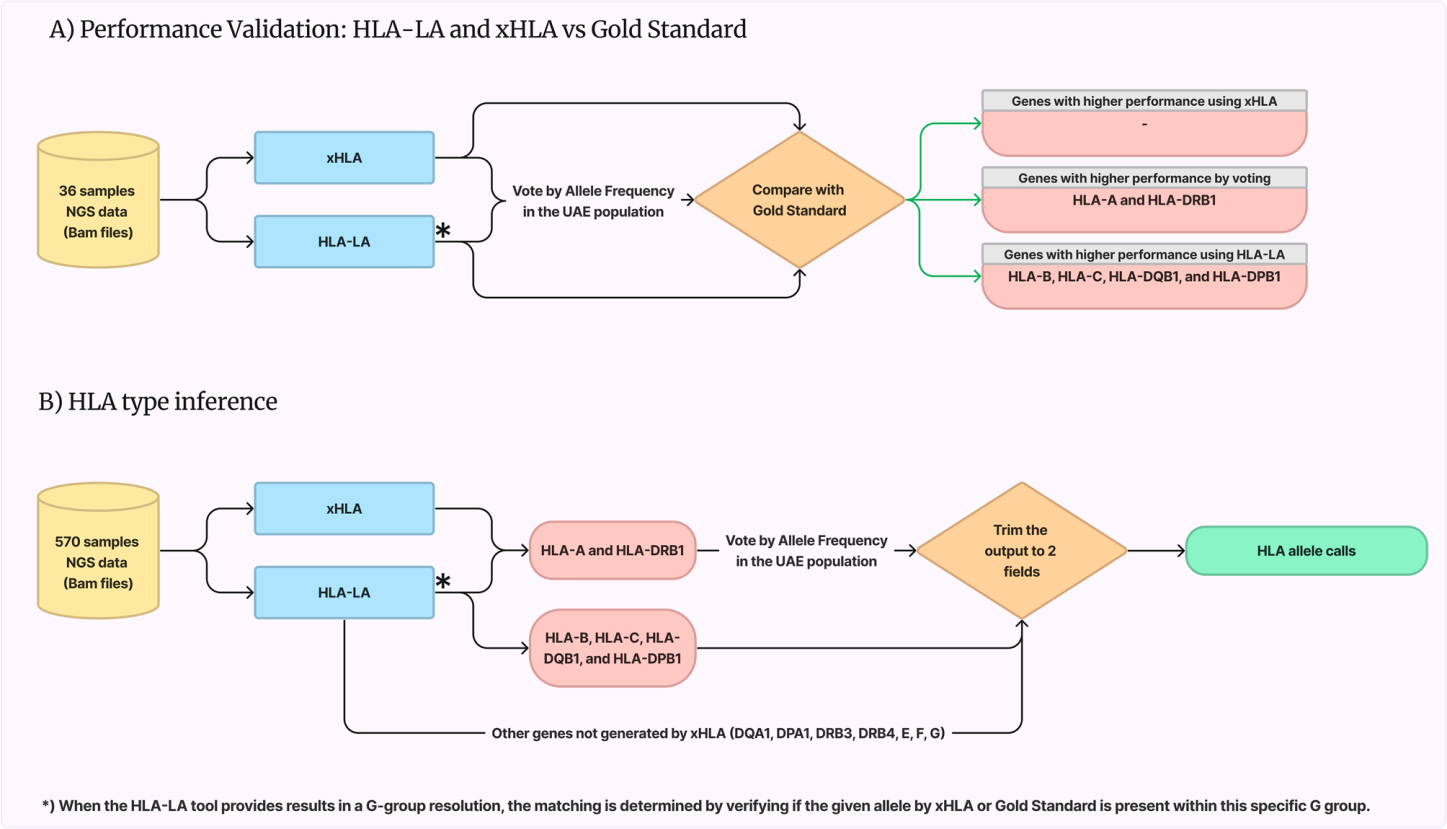
Workflow of the HLA alleles calling using bioinformatics tools. (**A**) Validation of the bioinformatics tools (i.e., HLA-LA and xHLA) performance versus gold standard targeted sequencing using 36 samples. HLA-LA showed higher accuracy in HLA-B, HLA-C, HLA-DPB1, and HLA-DQB1 genes. Voting by allele frequency showed higher in the HLA-A and HLA-DRB1 genes. (**B**) The used workflow for HLA alleles calling in the 570-sample cohort. *) When the HLA-LA tool reports group-based results (G group), the matching with the alleles from the gold standard or xHLA was done by checking if the G group contains the given allele. HLA-LA was used for the rest of the HLA class II and non-classical genes because xHLA does not process them. Detailed accuracies are listed in Supplementary Table S1 and Supplementary Fig. 1.

To evaluate the performance of the bioinformatic tools, results obtained from the xHLA and HLA-LA tools were compared with results obtained from the gold standard dataset (Fig. 1). When the HLA-LA tool reports group-based results (G group), the matching with the alleles from Omixon or xHLA was done by checking if the G group contains the given allele. For the definition of the HLA groups, IPD-IMGT/HLA Database v3.52.0 (http://hla.alleles.org/wmda/hla_nom_g.txt, date: 2023-04-17) was used^36,37^. Any missed values were excluded from the calculations. The accuracies of the tools for each HLA gene were recorded (Supplementary Table S1 and Supplementary Fig. 1). xHLA tool showed higher accuracy in the HLA-A and HLA-DRB1 genes. HLA-LA showed higher accuracy in HLA-B, HLA-C, HLA-DPB1, and HLA-DQB1 genes.

Next, further analysis was conducted to explore the potential enhancement of accuracy by combining the outputs of both tools and selecting alleles based on their allele frequencies (AF). The combining strategy involved examining the AF of the output alleles from both tools and selecting the two alleles with the highest AF in the UAE population^19^. In instances where there was a tie in the AF, higher priority was given to the allele that the tool had generated for the relevant gene. Also, the selection process prioritized the alleles that had a matched first field in both tools. For instance, if the two tools suggested four different alleles with two alleles matching in the first field, one of these two alleles would be selected based on the AF, irrespective of the AF of the remaining two mismatched alleles. The HLA-A and HLA-DRB1 genes showed enhancements in accuracy compared to the individual tools (i.e. xHLA and HLA-LA) (Supplementary Table S1 and Supplementary Fig. 1). HLA-LA was used for the rest of the HLA class II and non-classical genes because they are not processed by xHLA.

### Comparison of HLA calling from WGS and WES

To assess potential disparities or biases in HLA allele calling between WGS and WES, a comparison was made for each identified allele. The counts derived from WGS data were compared with those from the WES data. Fisher’s exact test was performed using the *fisher.test* function in R (v4.3.0) for every detected allele in the HLA genes: A, B, C, DQB1, DRB1, DQA1, DPA1, and DPB1.

### Comparison with other populations

The HLA allele frequencies were downloaded from the Allele Frequency Net Database (AFND, www.allelefrequencies.net)^38^. Only alleles with two or more fields were downloaded. The HLA allele frequencies were downloaded using the code available on GitHub as of July 5, 2023 (available at https://github.com/slowkow/allelefrequencies). Any reported allele in the G group format was converted to two fields by removing the third field^39^. After trimming the third and fourth fields, the frequencies were summed for any identical alleles in the same population and the same study.

Representative populations with more than 95 samples and classified as Gold for HLA-A, HLA-B, and HLA-DRB1 from each geographical region were selected from the AFND. The final list of populations (n = 99) is listed in Supplementary Table S11.

The POPTREE2 software^40^ was used for calculating the Fst (Fixation Index) and Gst (Gene Divergence) genetic divergence and drawing the Neighbor-joining (NJ) phylogenetic trees.

For double-clustered heatmaps, the Euclidean distances for each dimension of the AF matrix were calculated and then clustered using “Seaborn”; a Python statistical data visualization library based on matplotlib (version 0.12.2)^41^.

### Principal component analysis

The principal component analysis was performed for the HLA AFs in the current cohort and the 99 selected population. The computation of principal components was executed using the Python programming language (version 3.10.9) and the "Scikit-learn" machine learning library (version 1.2.1)^42^. To enhance the visual clarity of the population distribution on the plot, adjustments were made to the placement of scattered points using the Python library "adjustText". Exact placements are shown in the zoomed version of each PCA plot.

### Population genetic analysis

The degree of heterozygosity, and Guo and Thompson Hardy Weinberg equilibrium (HWE) at a locus-by-locus level were obtained using Python for Population Genomics (PyPop v.0.7.0)^43^. Slatkin's PyPop version of the Ewens-Watterson (EW) homozygosity test of neutrality evaluated HLA loci's natural selection effect. The test identified the normalized deviation of homozygosity (Fnd), which is the difference between observed and anticipated homozygosity divided by the square root of its variance.

### HLA haplotype estimation

Within the present cohort, HLA haplotypes were estimated using the Estimation-Maximization algorithm, which is implemented in the Hapl-o-Mat R package^44^.

### Genome-level homozygosity

BCFtools^45^ were used to call the regions of homozygosity (ROH) at the genome level in 313 WES UAE samples aligned to the reference genome hg38. Default settings were used. The allele frequency parameter was calculated based on the 313 WES samples. These samples were used to ensure the similarity of the analysis at the genome level,therefore, the rest of the samples were not included due to having a different sequencing technology or being aligned to different reference genomes (i.e., hg19). The percentage of the homozygosity at the genome level (i.e., autosomes only) was calculated by dividing the total length of the ROHs in the sample by the total length of the autosomes. Guided by Ceballos et al.^46^, K-means clustering (k = 2) was applied to the ROHs total length and ROHs number in the samples to divide samples into two clusters. One cluster contained the samples with smaller ROHs while the other cluster represented the samples with larger ROHs. This division aims to get a cluster that is enriched with possible consanguineous subjects.

## Results

### HLA allele frequency distribution in the UAE population

High-resolution (2-fields) HLA types were inferred for 570 unrelated healthy UAE nationals using HLA-LA and xHLA tools. Figure 1 illustrates the study design and pipeline used to obtain confident calling. To validate the output of the tools, we used thirty-six samples that were previously typed using a targeted NGS-based HLA sequencing strategy (considered as gold standard), in addition to HLA-LA and xHLA. Based on the performance of the individual tools and a combining strategy based on allele frequency, the HLA-LA tool for the HLA-B, HLA-C, HLA-DQB1, and HLA-DPB1 genes and the combining strategy for the HLA-A and HLA-DRB1 genes (Supplementary Table S1) were selected. HLA-LA was chosen for the remaining HLA class II and non-classical genes, as they are not processed by xHLA.

Supplementary Tables S2, S3, and S4 present the counts and frequencies of HLA class I, class II, and non-classical. The most frequent HLA class I alleles in each locus were HLA-A*02:01 (14.035%), HLA-B*51:01 (9.211%), and HLA-C*04:01 (14.825%). On the other hand, the most frequent HLA class II alleles in each locus were HLA-DRB1*03:01 (16.053%), HLA-DQA1*01:02 (28.333%), HLA-DQB1*02:01 (26.14%), HLA-DPA1*01:03 (64.123%), and HLA-DPB1*04:01 (30.439%).

A significant deviation from Hardy–Weinberg Equilibrium (HWE) was observed in all HLA loci except HLA-A and HLA-DPB1 (Supplementary Table S5). From the Ewens-Watterson (EW) homozygosity test, a significant excess of homozygosity in HLA-DPA1 and HLA-DPB1 loci was identified by positive normalized deviation of homozygosity (Fnd) of 1.1030 and 2.0197, respectively (Supplementary Table S6). In contrast, HLA-A, HLA-C, HLA-B, HLA-DRB1, HLA-DQA1, and HLA-DQB1 loci exhibited negative Fnd values, indicating less homozygosity than expected.

From the 4-locus (HLA-A-B-DRB1-DQB1) haplotype frequencies (HF), the most frequent haplotype in this cohort was: HLA-A*26:01 ~ B*08:01 ~ DQB1*02:01 ~ DRB1*03:01 (HF: 1.85%). The top 400 haplotype frequencies (HF) are listed in Supplementary Table S7.

### Associations with diseases

It is interesting to note that among the most frequent alleles (AF > 0.05), were variants that are strongly associated with different autoimmune diseases as well as drug hypersensitivity reactions (Supplementary Fig. 2A). For example, HLA-B*51:01, the most common HLA-B allele (9.21%), is known to be associated with Behçet's Disease^47^. The HLA-DRB1*03:01 allele, with a prevalence of 16.05%, has been associated with increased susceptibility to Type 1 diabetes^48^, Rheumatoid arthritis^49^, and Systemic lupus erythematosus (SLE)^50^. The HLA-DRB1*15:01 allele has been identified as a potential factor in the development of Multiple Sclerosis (MS)^51^, with a prevalence of 6.14%. Notably, the HLA-DQB1*02:01 allele and the most prevalent HLA-DQB1 gene variant in the studied population is widely recognized for its association with Celiac Disease^52^.

### Associations with drug toxicity

Several detected HLA alleles have been linked to pharmacogenomics (Supplementary Fig. 2B). For instance, HLA-C*06:02, the second most prevalent allele (14.035%), is associated with hypersensitivity responses to sulfamethoxazole/trimethoprim^53^. Moreover, the allele HLA-DPB1*03:01 (8.772%), which has been linked to sensitivity to aspirin^54^, was shown to be the third most prevalent allele. On the other hand, several alleles that have been linked to pharmacogenomics markers of HLA-A1 evidence levels have low frequencies in our cohort. For example, HLA-B*58:01, which is linked to allopurinol hypersensitivity^55^, was detected with a frequency of 0.042. Similarly, the allele HLA-A*31:01, associated with carbamazepine hypersensitivity^56^, exhibited a frequency of 2.9%. The allele HLA-B*57:01 which is linked to the sensitivity to abacavir and flucloxacillin^57,58^, and the allele HLA-B*15:02 which is linked to the sensitivity to carbamazepine^59^, phenytoin^60^, sulfamethoxazole/trimethoprim^53^, lamotrigine, oxcarbazepine, and antiepileptics^61^, were observed at notably lower frequencies of 0.53% and 0.44%, respectively.

### Comparative analysis with other populations

**Figure 2:**
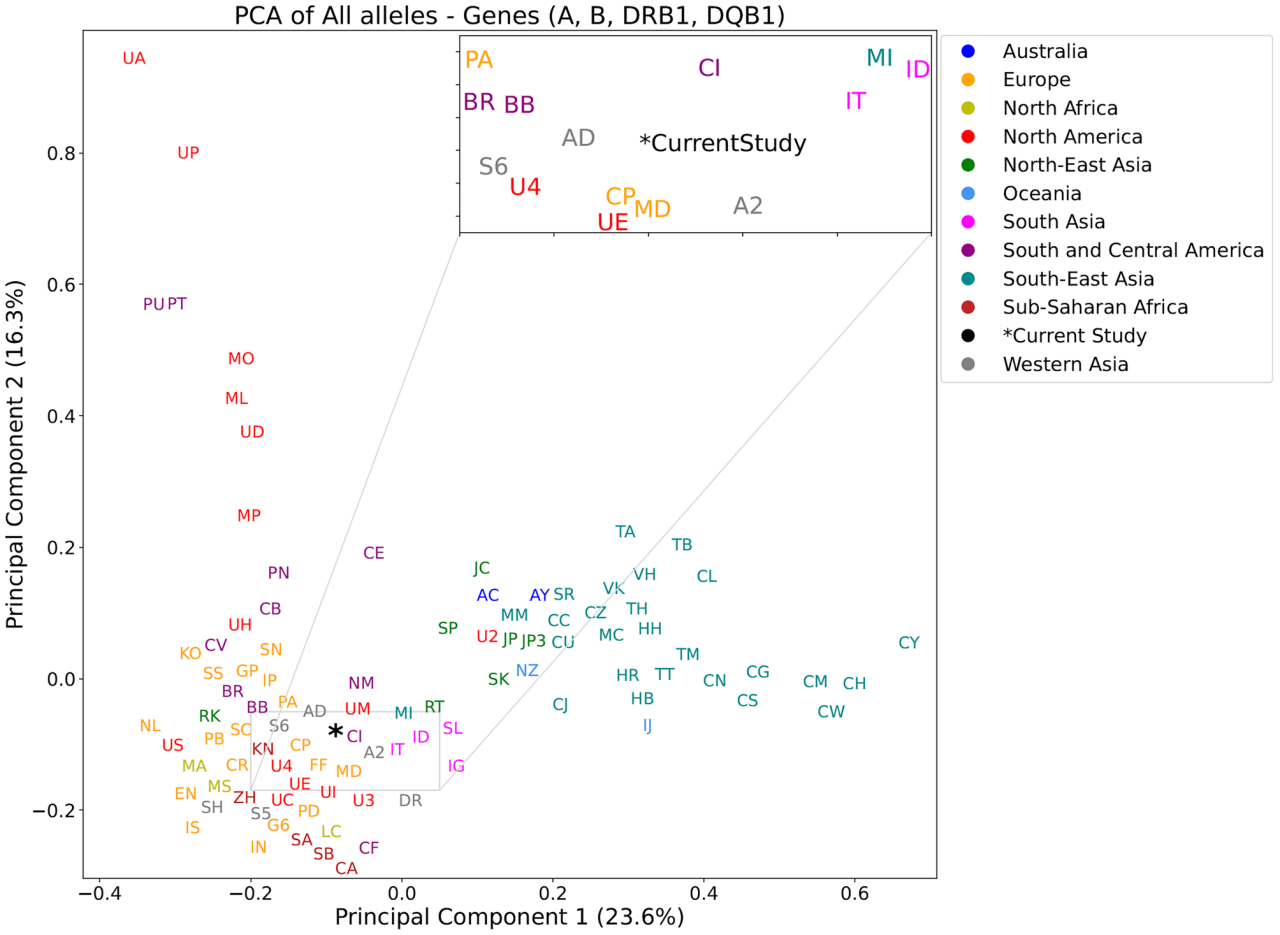
Principal Component Analysis (PCA) for 100 populations including the current study. The PCA is based on the AFs of the HLA alleles in HLA-A, HLA-B, HLA-DQB1, and HLA-DRB1 genes. Population names are colored based on the region. The zoom panel shows the current study and its adjacent populations. The full names of the populations are listed in Supplementary Table S11.

Considering the high level of polymorphism in HLA-A, -B, HLA-DRB1, and HLA-DQB1 genes, they represent highly informative markers. Hence, their allelic variations were used for conducting Principal Component Analysis (PCA) and phylogenetic tree for 100 world population datasets retrieved from the AFND, including the current cohort (Fig. 2). The current UAE cohort is located at the intersections of different population clusters including Western Asia, North Africa, and Europe, illustrating a rich ancestral influence that provides perspective on the region’s historical interactions, as emphasized previously^33^. The PCA plot also confirms the link between HLA allele frequency and geography as previously reported^13^.

**Figure 3:**
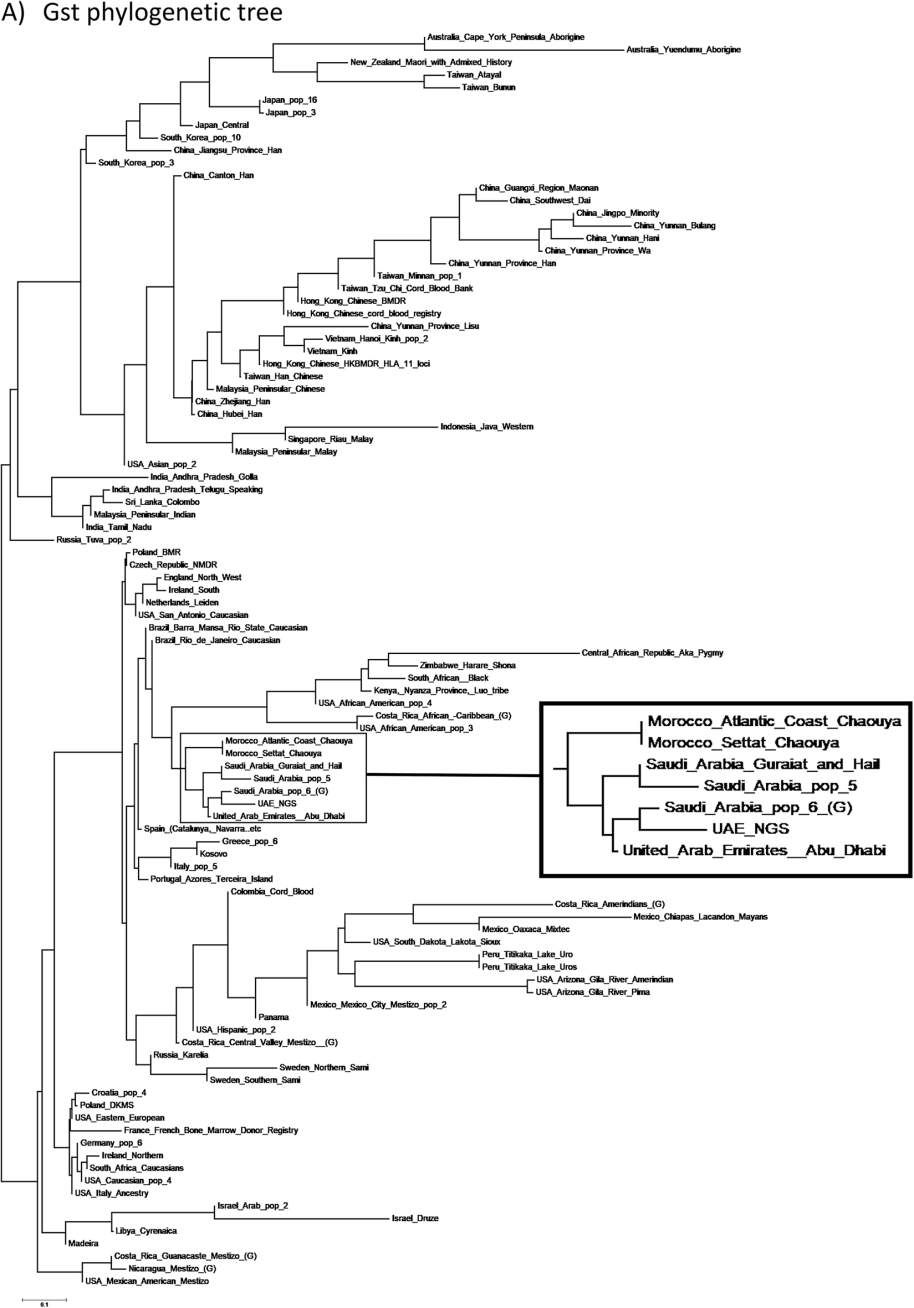

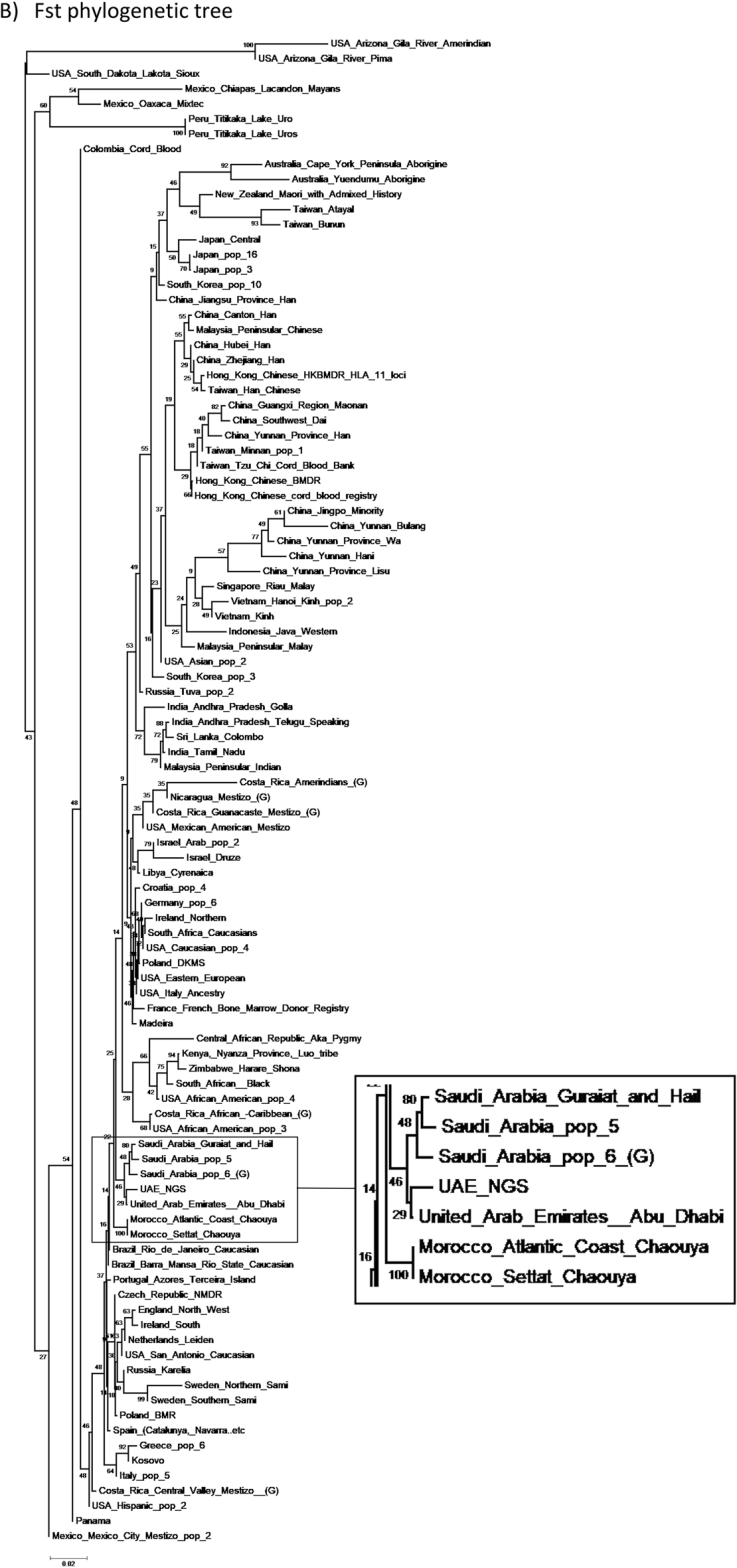
Phylogenetic tree illustrating (**A**) Gst and (**B**) Fst comparisons between the present study and 99 other chosen populations. The tree was constructed using the HLA-A, HLA-B, HLA-DQB1, and HLA-DRB1 genes. The tree was created with the Neighbor-Joining (NJ) method using the Poptree2 software. The zoom panel shows the current study and its adjacent populations.

The same datasets were used to build a phylogenetic tree using both Fst (Fixation Index) and Gst (Gene Divergence) genetic divergence measures using POPTREE2 (Fig. 3). Specifically, the analysis revealed the distinct patterns of the genetic variations and the relative relationship among these populations. The Fst corrected measure is more sensitive with bi-allelic markers, while the Gst is better at handling high levels of intra-population diversity with multi-allelic markers. Both metrics were employed to provide a robust description of the global population structure. From the Fst-corrected phylogenetic tree, the current cohort clustered closely with previous UAE, three different Saudi Arabian, and two different Moroccan (North African) datasets, further providing evidence of the accuracy of the HLA type calling method employed herein. Relatively similar clustering was obtained from the Gst phylogenetic tree, with further clustering of these populations with Central and Southern African populations. Overall, both the Fst and Gst phylogenetic trees provided slightly different qualitative results when compared to the PCA, which is not uncommon due to variations in the methodology.

**Figure 4:**
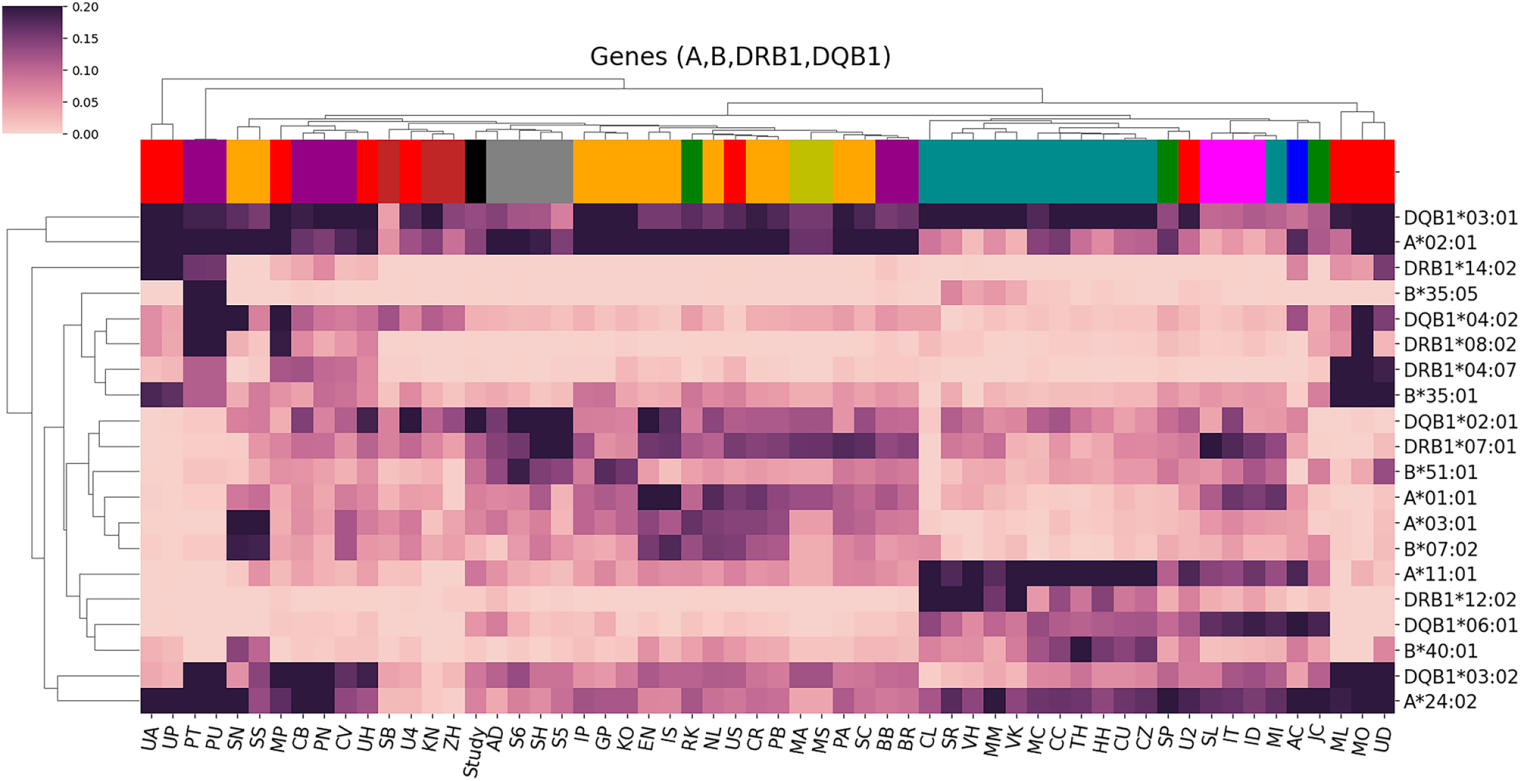
Allele frequency heatmap for the HLA-A, HLA-B, HLA-DQB1, and HLA-DRB1 genes in 100 populations including the current cohort. The heatmap is double clustered using Euclidean distance. We removed alleles with variance < 0.001 across all populations and excluded populations with a total allele frequency sum < 0.9. The full names of the populations are listed in Supplementary Table S11.

In agreement, from the double clusters frequencies heatmaps (Fig. 4), the alleles that are common among the current study, the Abu Dhabi UAE dataset, and the three Saudi datasets included HLA-A*02:01, HLA-B*51:01, HLA-DQB1*02:01, HLA-DQB1*03:01, HLA-DQB1*03:02, and HLA-DRB1*07:01 among others. Heatmaps for individual HLA genes are available in Supplementary Figs. S3–S10. Thirty-two rare alleles (AF < 0.003) were detected in our cohort, absent in the other 99 selected populations. Most of these alleles (n = 20) are located in HLA class II genes (Supplementary Table S8).

### Comparative analysis between the detected HLA alleles and the published UAE dataset

A detailed comparison between our cohort and the sole Gold standard submission from UAE in the AFND titled “United Arab Emirates Abu Dhabi'' (n = 52 samples), obtained from Arnaiz-Villena et al.^29^ was performed. The comparison focused on the common genes between this study and the submission: HLA-A, HLA-B, HLA-C, HLA-DQB1, HLA-DRB1, and HLA-DQA1. This study identified 132 alleles that weren't present in the database submission, with the highest AF being 2.4% for HLA-B*41:01. On the other hand, the database submission listed 6 unique alleles that absent in our data: HLA-A*24:11, HLA-A*24:17, HLA-B*15:220, HLA-C*07:06, and HLA-C*17:03, where all these alleles have an AF of 0.96%, and DQB1*03:19 with an AF of 3.9% Notably, the HLA-DQA1 gene did not exhibit any unique alleles in both datasets.

### HLA allele frequencies in WGS and WES samples

The statistical analysis for the AFs in HLA gene class I and class II showed no significant difference for the AFs obtained from WGS and WES samples (Supplementary Table S9). The maximum AF difference was observed in the DPA1 gene for the allele DPA1*01:03 with a difference of 8.4% (70% in WGS vs. 62% in WES). All other genes showed 5% or less as a maximum difference in the AFs between WGS and WES samples. As expected, rare alleles were more frequently observed in WES samples compared to WGS samples, owing to the larger sample size of WES in this study.

### HLA allele and genome-wide homozygosity

The UAE population is known for its high rate of consanguineous marriages^62^, and consanguineous subjects are characterized by a high level of genome-wide homozygosity^46^. For that, an additional analysis was performed where 313 WES samples were used to assess the homozygosity at both the genome-wide and HLA allele levels. Due to the lack of information about the consanguinity status of each sample, the samples were divided into two groups based on the regions of homozygosity (ROH) at the genome level (Supplementary Fig. 11) and named high-ROH and low-ROH clusters. This division aims to get a cluster with larger ROHs that is enriched with subjects who are more likely to be the offspring of a consanguineous marriage. As expected, the HLA genes homozygosity was higher in high-ROH samples compared to low-ROH samples for all HLA genes. However, only HLA-B and HLA-C genes showed statistically significant differences (Supplementary Table S9). Interestingly, the levels of homozygosity at the HLA genes were higher than those observed at the autosomal level in both high-ROH and low-ROH samples across all HLA genes (Supplementary Fig. 12).

## Discussion

This study provides a comprehensive analysis of the DNA sequences of classical HLA genes in the UAE population using next-generation sequencing (NGS). Notably, this study features the largest UAE cohort used to study HLA alleles of the diverse population that lives in the South Eastern tip of the Arabian peninsula, unveiling specific characteristics with potential implications in disease association studies and histocompatibility matching. The inclusion of 119 WGS samples from the study by Daw Elbait et al.^33^ was to ensure that a considerable level of diversity was represented in this research. These 119 unrelated samples were specifically chosen from > 1,000 UAE nationals^63^ to account for diversity and consider admixture calculations^33^.

Intra-population and inter-population analyses confirm an admixed population in the UAE population. The similarity in HLA allele frequencies with other Arabian populations supports some degree of shared genetic heritage. Despite the relatively significant geographical distances between the Arabian populations near the UAE (West Asia) with two different Moroccan (North African) datasets, the populations cluster closely. Yet, the unique alleles and haplotypes identified in this study highlight the need for population-specific databases to improve the chances of histocompatibility matching for transplantation. The discovery of 132 alleles, not previously reported in the “United Arab Emirates Abu Dhabi'' database^29^ further emphasizes the importance of the current study and underscores the need for future larger studies to further characterize HLA allele repertoire in the population. Further studies are also required to investigate the potential implications of these alleles in health and transplantation. Access to allelic frequency information specific to the studied population enhances the precision of HLA findings and feature research. This is particularly the case when bioinformatic tools provide differing allele determinations, as seen with the HLA-A and HLA-DRB1 loci in this research.

The frequencies and distributions of HLA alleles associated with drug toxicity and some diseases from this study were similar to previous reports^29,64^. However, noteworthy differences were also identified. For instance, the HLA-DQB1*02:01 allele, linked to Celiac Disease^52^, had an allele frequency (AF) of 26.14%, compared with 15.4% in the "United Arab Emirates Abu Dhabi" dataset^29^. While the difference in AF lacks statistical significance, it elevates the AF to above the average in diverse global regions (Supplementary Fig. 2). These findings align with the high prevalence of Celiac Disease in the UAE and Arabian Peninsula region, as compared to the worldwide population^65,66^. Nevertheless, a disparity in the allelic frequency for the HLA-DQB1*02:01 was evident in datasets originating from the same country, such as Saudi Arabia (AFND datasets: Saudi Arabia pop 3 and Saudi Arabia Guraiat and Hail, 46% vs 21%), Tunisia (AFND datasets: Tunisia pop 2 and Tunisia Ghannouch, 43% vs 19.5%), and Algeria (AFND datasets: Algeria Oran and Algeria pop 2, 32.8% vs 23.8%). This may be due to differences in the sample selection criteria in each study or due to the sensitivity of the method used for this given allele. Saudi Arabia pop 6 and Algeria pop 2 datasets showed the closest AF among Arab populations (29.9% and 23.8% respectively) to the cohort studied here. Notably, the high AF of the HLA-DQB1*02:01 allele places it in the top frequent HLA haplotypes observed in this study (Supplementary Table S7). A noteworthy illustration is that over 11% of the samples exhibit the DQA1*05:01 ~ DQB1*02:01 haplotype, which has implications not only for celiac disease risk but also demonstrates varying risk levels based on the presence of the alleles DRB3*01:01:02 or DRB3*02:02:01^67^. This distinction indicates that different haplotypes confer distinct risks for celiac disease. This underscores the significance of HLA alleles not just at the individual gene level but also within the context of haplotypes^68,69^.

The UAE, similar to the other countries in Middle East, is known to have high percentage of consanguinity (39%-54%) compared to North America, Europe and Australia (< 5%)^62,70^. Consanguineous marriages may lead to genetic consequences, especially concerning the increased likelihood of homozygosity which has an impact on susceptibility to autosomal recessive diseases. Homozygosity of HLA alleles also holds prognostic value in immunotherapy, where treatment efficacy can be closely tied to the interaction between the immune system and specific molecular targets^71^. Homozygosity for favorable genetic markers could signify a more robust and/or consistent immune response against targeted antigens. The observed level of HLA alleles homozygosity in this study correlates with previous findings in other Arabian populations^25^. The findings in this study reflect an increase in HLA genes homozygosity among samples with higher genome-wide homozygosity levels, especially for HLA class I genes. Additionally, the higher level of homozygosity observed for HLA genes compared to autosomal genes, regardless of the genome-wide ROH levels, raises some intriguing questions. It warrants a deeper investigation to discern if this is due to the unique selective pressures on the HLA genes, or other factors yet to be elucidated. This highlights the need for further studies to investigate these findings in the context of immunotherapy and other clinical implications. The absence of consanguinity information for each sample was a limitation in this study. As an alternative approach, the samples were divided into low- and high- runs of homozygosity (ROH). This stratification aims to an enriched group that is specifically comprised of individuals with consanguineous relationships^46,72^.

The use of NGS affords the opportunity to investigate the HLA at a high-resolution level without the need to perform any imputations, a challenge faced in array-based analysis. However, as demonstrated herein, there is still a need to establish gold standards to allow for the assessment of the bioinformatics tools utilized for analysis. Nonetheless, despite the anticipated biases in allele calling between WGS and WES techniques, the analyses in this study have revealed minimal discrepancies in allele frequencies between the two methods. This consistency lends further credibility to the findings of this study and argues for the reliability of NGS-based HLA typing.

This study expands on existing information with data collected here in the search of new knowledge, aimed at improving clinical outcomes, and advancing our understanding of human genetics. However, it is essential to acknowledge certain limitations of the methodology used. Although both HLA typing tools have been validated using a defined gold-standard HLA typing method, those tools rely heavily on publicly available databases in which the Arabian genome is underrepresented. The distinct genetic architecture of the Arabian genome with its unique allele distribution may lead to misclassifications of alleles or neglect of region-specific, rare variations when using these reference-based tools.

Finally, based on the analysis of HLA genes in the UAE population reported here, several key recommendations can be proposed. It is important to continuously enrich HLA databases, given the discovery of 132 previously unreported variants. The establishment of population-specific HLA databases is crucial to enhance transplantation accuracy. Integrating pharmacogenomic data into clinical practice can be used to mitigate drug hypersensitivity reactions associated with specific HLA alleles. The elevated HLA homozygosity due to consanguinity underscores the need for genetic counseling in some communities. Establishing a gold standard for evaluating HLA typing methods and analysis is vital for reproducibility and reliability. Finally, fostering international collaboration in population-based genetics research will broaden our understanding of genetic diversity's impact on healthcare, benefiting not only the UAE but also global populations. Further work is required to expand the work described here to incorporate the definition of MHC haplotypes. The polymorphic nature of the genes in these haplotypes contributes to the incredible diversity in antigen presentation, facilitating a robust immune response. Therefore, MHC haplotypes are integral to transplantation compatibility, autoimmune disease susceptibility, and overall immune system function.

### Supplementary Information

Supplementary Information 1.

Supplementary Information 2.

## Data Availability

The dataset supporting the conclusions drawn from this cohort is accessible in the supplementary materials. Individual-level data are restricted and can be obtained from the corresponding author upon a reasonable request.
