# Supplementary material for: Investigating the genetic makeup of the major histocompatibility complex (MHC) in the United Arab Emirates population through next-generation sequencing
Source: Sci Rep. 2024 Feb 9;14:3392. doi: 10.1038/s41598-024-53986-1 (PMC10858242; doi:10.1038/s41598-024-53986-1)

Investigating the Genetic Makeup of the Major Histocompatibility Complex (MHC) in the United Arab Emirates Population through Next-Generation Sequencing

Nour al dain Marzouka[1&], Halima Alnaqbi[1&], Amira Al-Aamri[1], Guan Tay[2,3], Habiba Alsafar[1,4,5,*]

[1] Center for Biotechnology, Khalifa University of Science and Technology, Abu Dhabi, United Arab Emirates.
[2] Division of Psychiatry, Faculty of Health and Medical Sciences, Medical School, The University of Western Australia, Crawley WA, Australia.
[3] School of Medical and Health Sciences, Edith Cowan University, Joondalup WA, Australia.
[4] College of Medicine and Health Sciences, Khalifa University of Science and Technology, Abu Dhabi, United Arab Emirates.
[5] Department of Biomedical Engineering, Khalifa University of Science and Technology, Abu Dhabi, United Arab Emirates.

&: equal contribution
*Corresponding author
Editorial corresponding author: Habiba Alsafar: Habiba.alsafar@ku.ac.ae

- **This file contains Supplementary Figures 1-12.**
- **The supplementary Excel file contains Supplementary Tables S1-S11.**

Supplementary Figure 1: Bioinformatic tools versus Gold Standard. The bar plot shows the accuracy of the HLA-LA and xHLA, and the voting by allele frequency versus gold standard. Based on the accuracies, we selected the HLA-LA tool for the HLA-B, HLA-C, HLA-DQB1, and HLA-DPB1 genes and the combining strategy for the HLA-A and HLA-DRB1 genes. HLA-LA was used for the rest of the HLA class II and non-classical genes because xHLA does not process them.

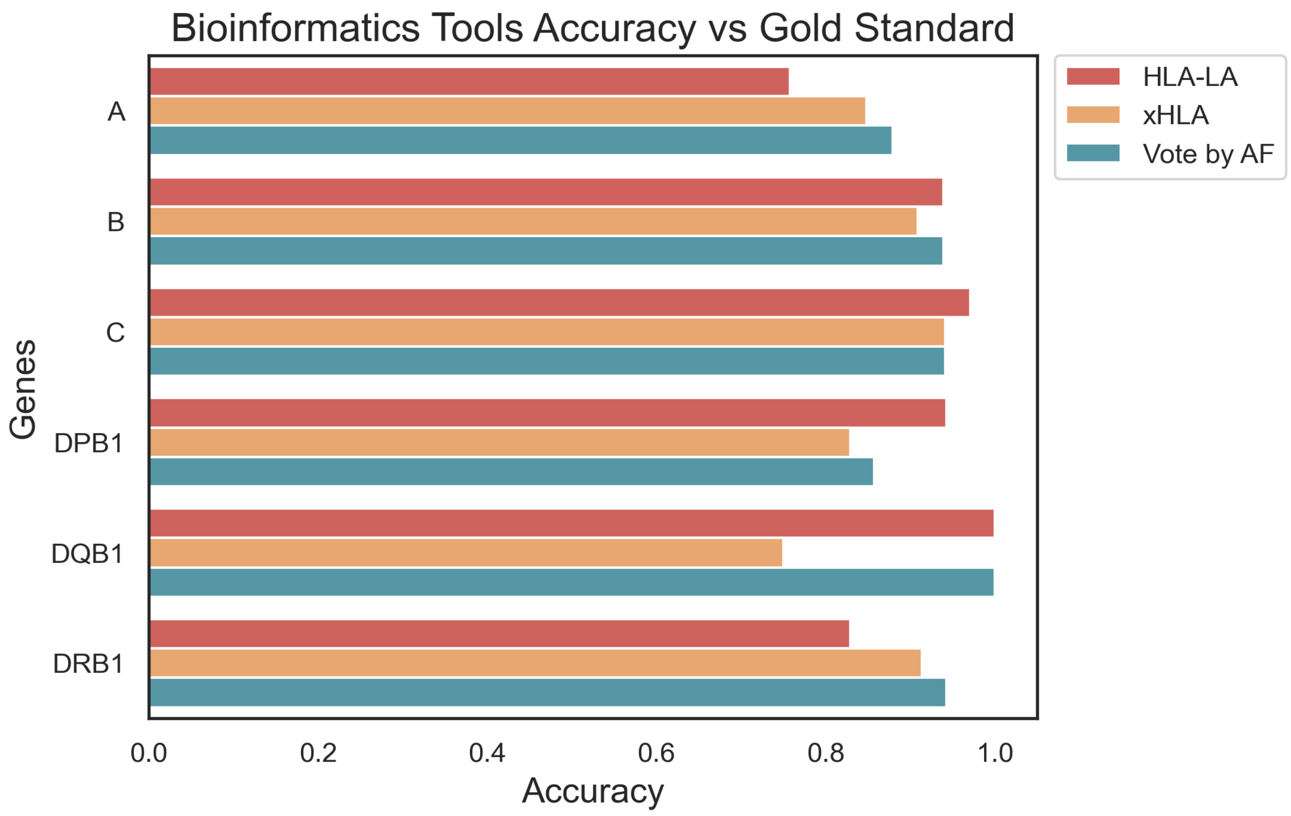

Supplementary Figure 2: Prevalence of Specific HLA Alleles with Clinical Relevance. A) HLA alleles linked to particular diseases. B) Most common HLA alleles in this study group known to relate to drug toxicity. The bar charts represent the present study group, the previously published Abu Dhabi group, and the mean allele prevalence in various global regions. The detailed populations can be found in Supplementary Table S11. Study: Current cohort, Abu Dhabi: AD, WA: Western Asia, NEA: North-East Asia, SA: South Asia, SEA: South-East Asia, EU: Europe, SCA: South and Central America, NA: North America, NAF: North Africa, SAF: Sub-Saharan Africa, AU: Australia, OC: Oceania.

A) HLA alleles linked to particular diseases.

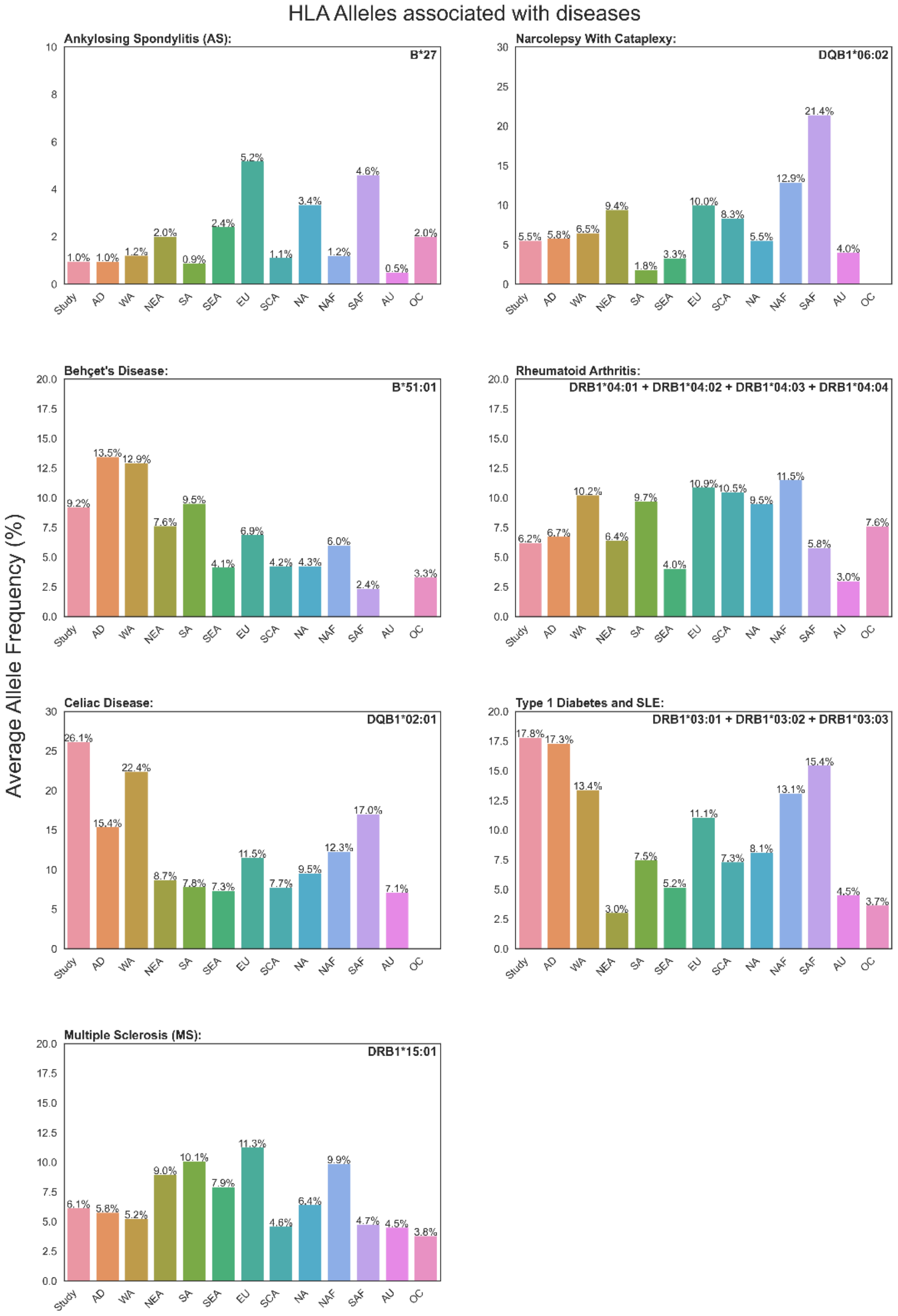

B) Most common HLA alleles in this study group known to relate to drug toxicity.

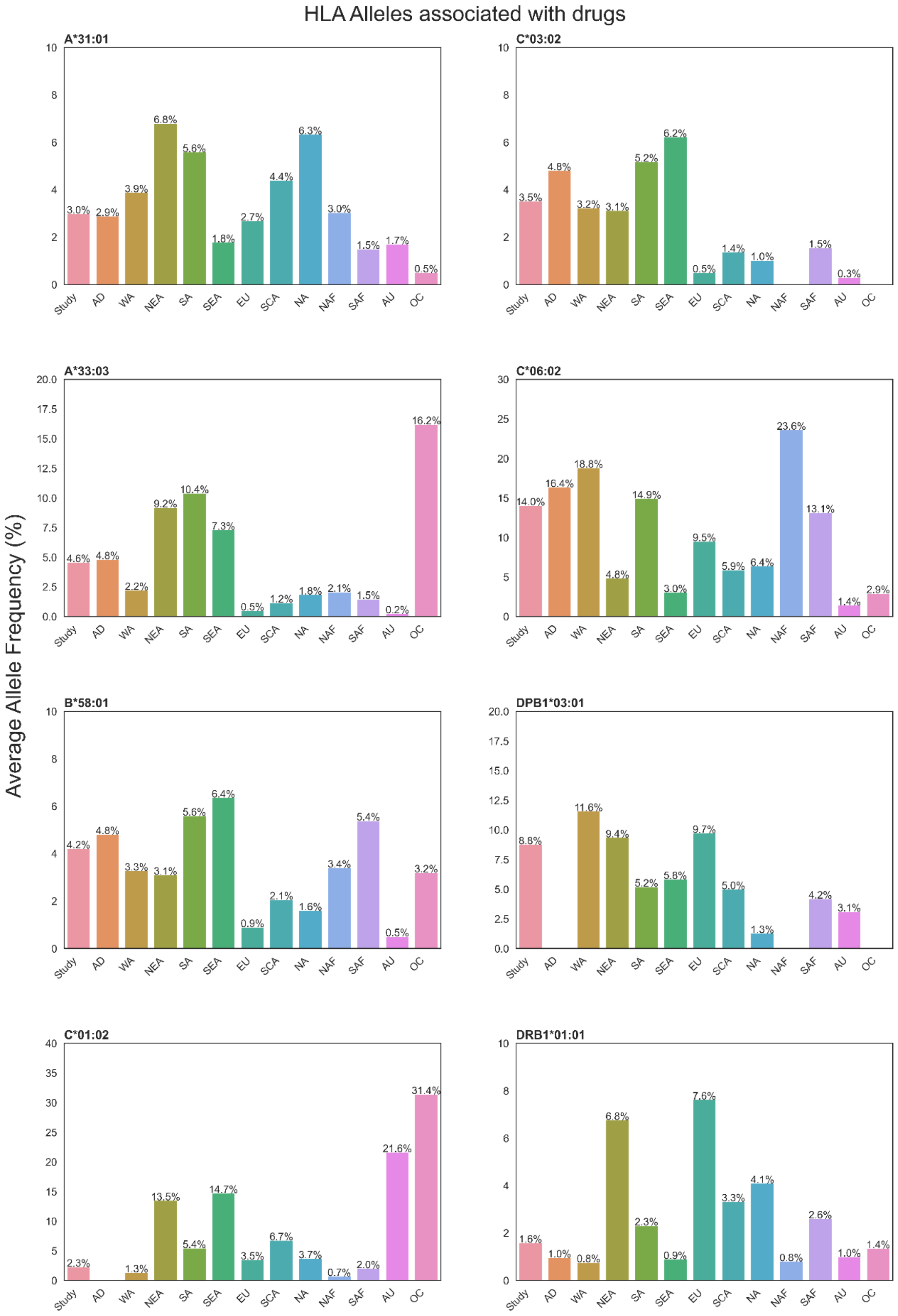

Supplementary Figure 3: Allele frequency heatmap for HLA-A genes in 100 populations including the current cohort. The heatmap is double clustered using Euclidean distance. We removed alleles with variance < 0.001 across all populations and excluded populations with a total allele frequency sum < 0.9. Only the populations shown in Figure 4 are included here for a comparable view. The full names of the populations are listed in Supplementary Table S11.

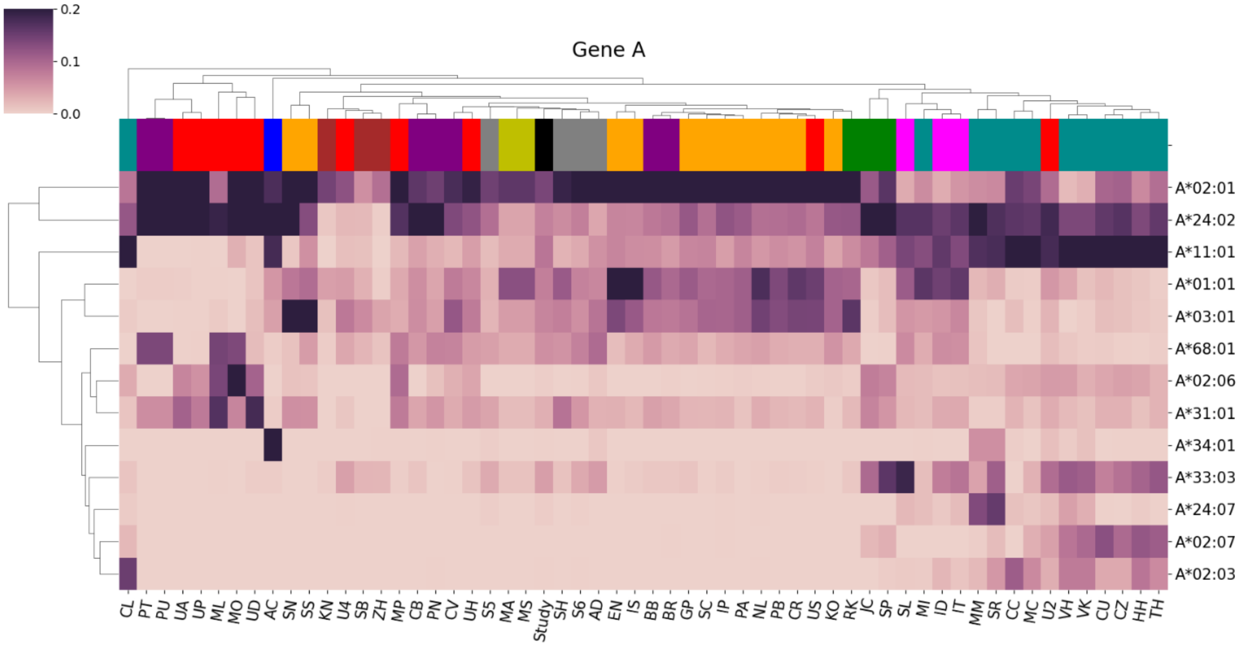

Supplementary Figure 4: Allele frequency heatmap for HLA-B genes in 100 populations including the current cohort. The heatmap is double clustered using Euclidean distance. We removed alleles with variance < 0.001 across all populations and excluded populations with a total allele frequency sum < 0.9. Only the populations shown in Figure 4 are included here for a comparable view. The full names of the populations are listed in Supplementary Table S11.

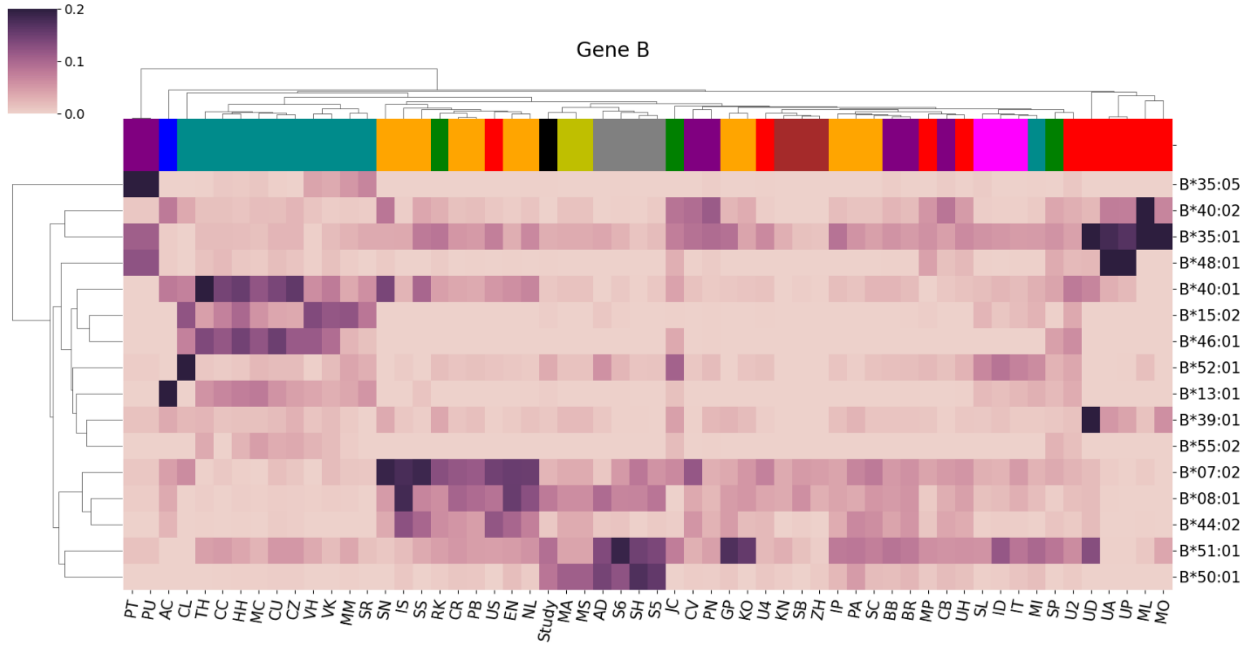

Supplementary Figure 5: Allele frequency heatmap for HLA-C genes in 100 populations including the current cohort. The heatmap is double clustered using Euclidean distance. We removed alleles with variance < 0.001 across all populations and excluded populations with a total allele frequency sum < 0.9. Only the populations shown in Figure 4 are included here for a comparable view. The full names of the populations are listed in Supplementary Table S11.

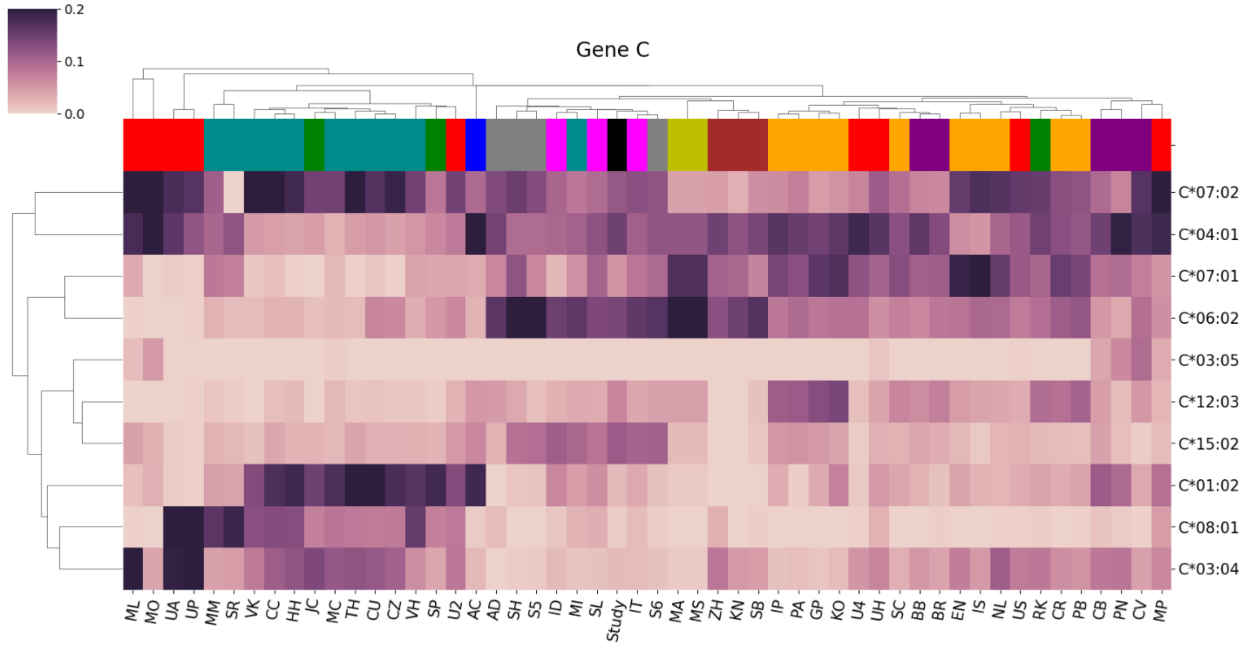

Supplementary Figure 6: Allele frequency heatmap for HLA-DRB1 genes in 100 populations including the current cohort. The heatmap is double clustered using Euclidean distance. We removed alleles with variance < 0.001 across all populations and excluded populations with a total allele frequency sum < 0.9. Only the populations shown in Figure 4 are included here for a comparable view. The full names of the populations are listed in Supplementary Table S11.

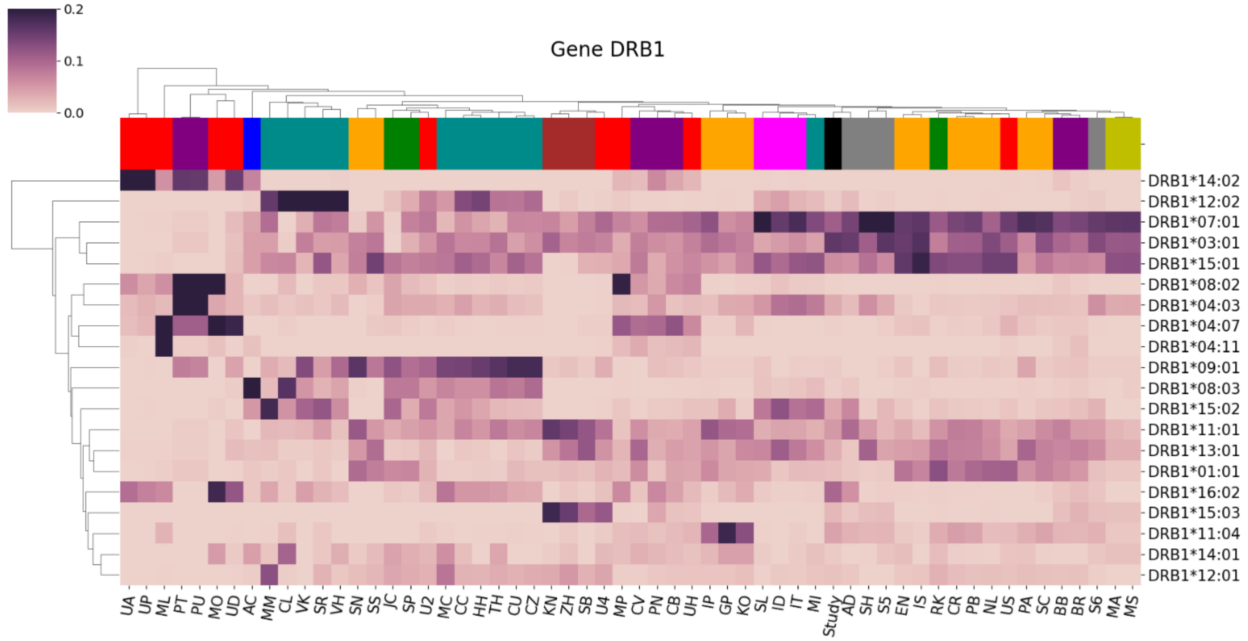

Supplementary Figure 7: Allele frequency heatmap for HLA-DQA1 genes in 100 populations including the current cohort. The heatmap is double clustered using Euclidean distance. We removed alleles with variance < 0.001 across all populations and excluded populations with a total allele frequency sum < 0.9. Only the populations shown in Figure 4 are included here for a comparable view. The full names of the populations are listed in Supplementary Table S11.

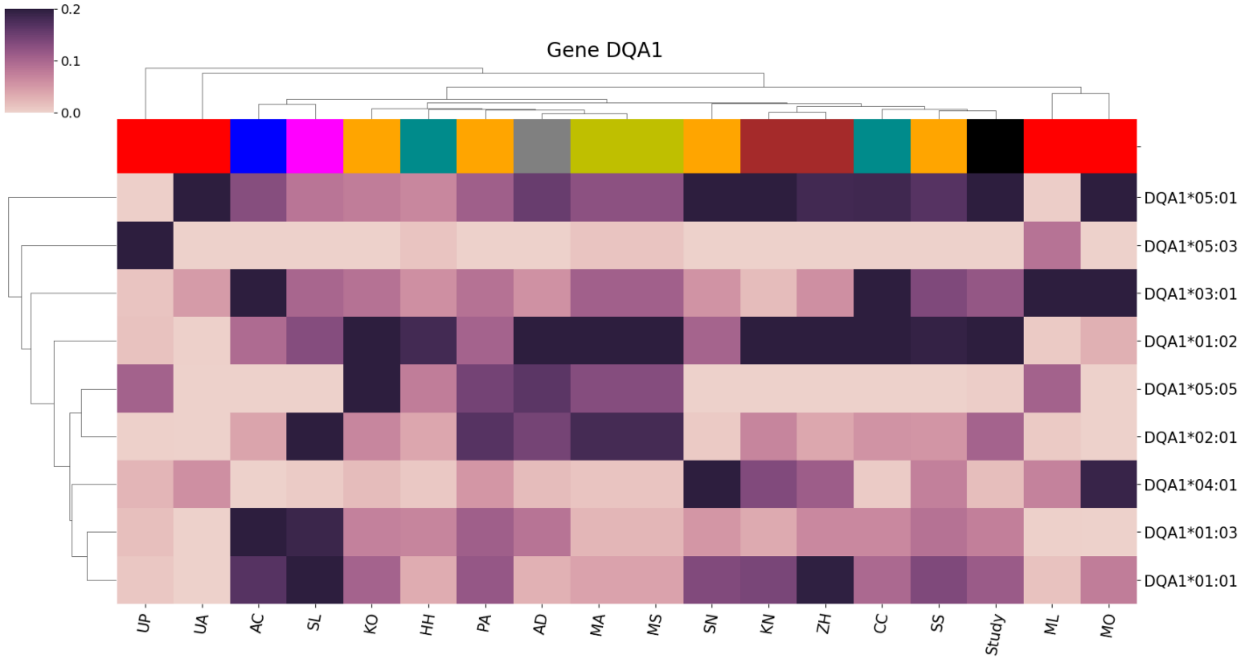

Supplementary Figure 8: Allele frequency heatmap for HLA-DQB1 genes in 100 populations including the current cohort. The heatmap is double clustered using Euclidean distance. We removed alleles with variance < 0.001 across all populations and excluded populations with a total allele frequency sum < 0.9. Only the populations shown in Figure 4 are included here for a comparable view. The full names of the populations are listed in Supplementary Table S11.

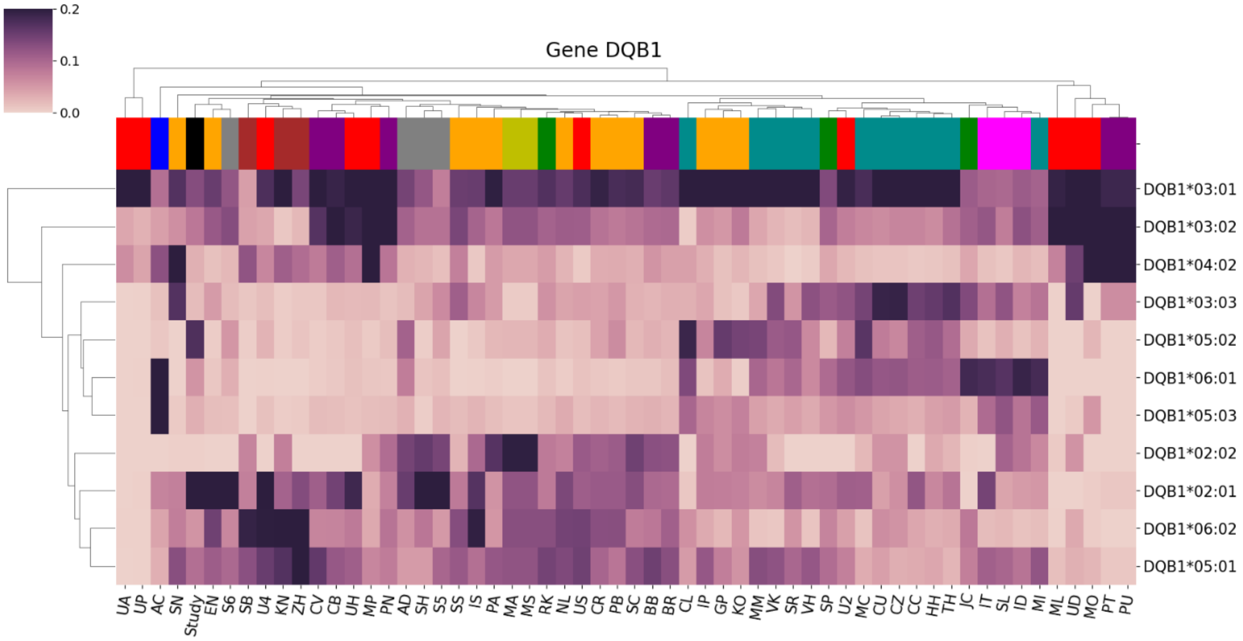

Supplementary Figure 9: Allele frequency heatmap for HLA-DPA1 genes in 100 populations including the current cohort. The heatmap is double clustered using Euclidean distance. We removed alleles with variance < 0.001 across all populations and excluded populations with a total allele frequency sum < 0.9. Only the populations shown in Figure 4 are included here for a comparable view. The full names of the populations are listed in Supplementary Table S11.

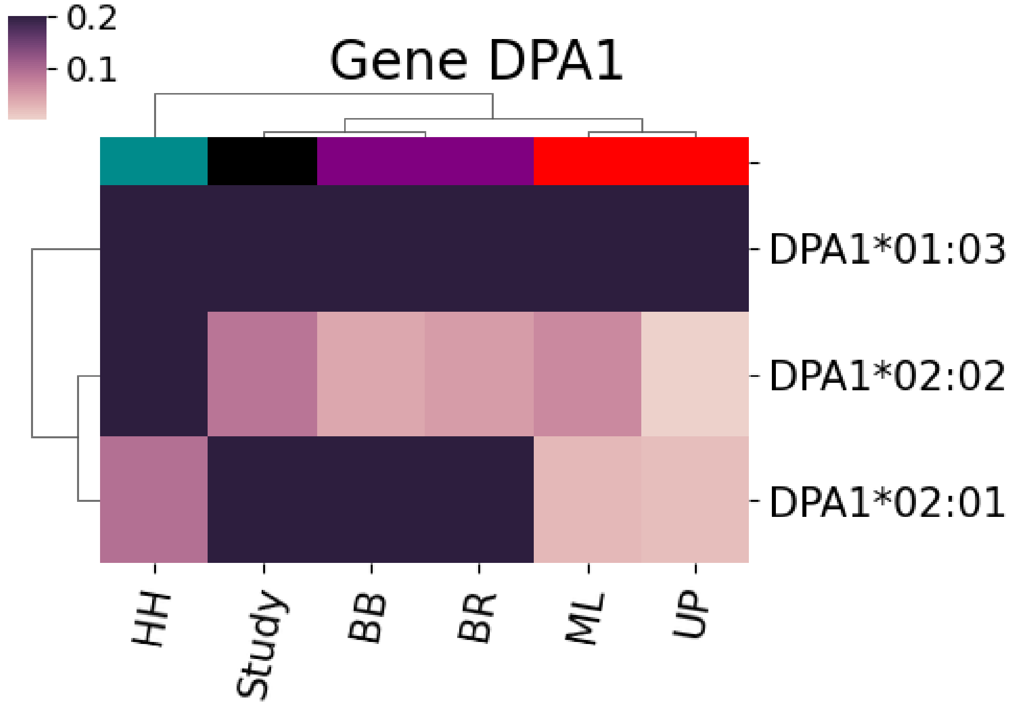

Supplementary Figure 10: Allele frequency heatmap for HLA-DPB1 genes in 100 populations including the current cohort. The heatmap is double clustered using Euclidean distance. We removed alleles with variance < 0.001 across all populations and excluded populations with a total allele frequency sum < 0.9. Only the populations shown in Figure 4 are included here for a comparable view. The full names of the populations are listed in Supplementary Table S11.

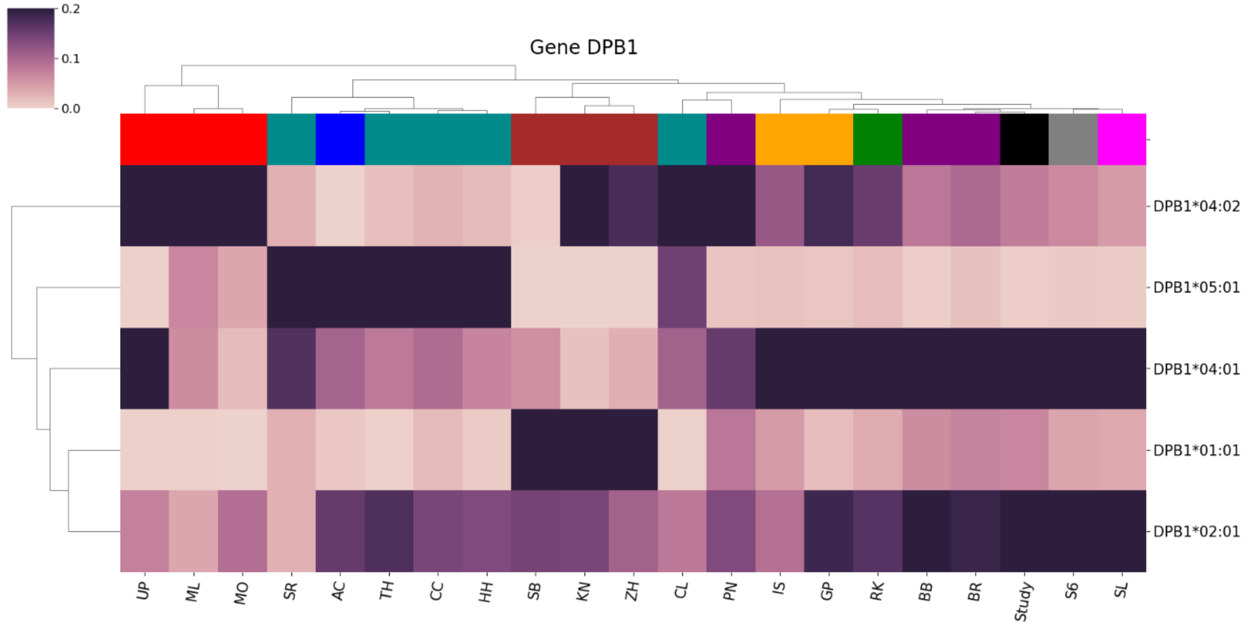

Supplementary Figure 11: K-means clustering for 313 whole exome samples based on regions of homozygosity (ROH) length and numbers in each sample. The blue cluster represents samples with high levels of ROH, while the yellow cluster contains the samples with lower levels of ROH. This division aims to get a cluster (i.e., blue cluster) that is enriched with possible consanguineous subjects.

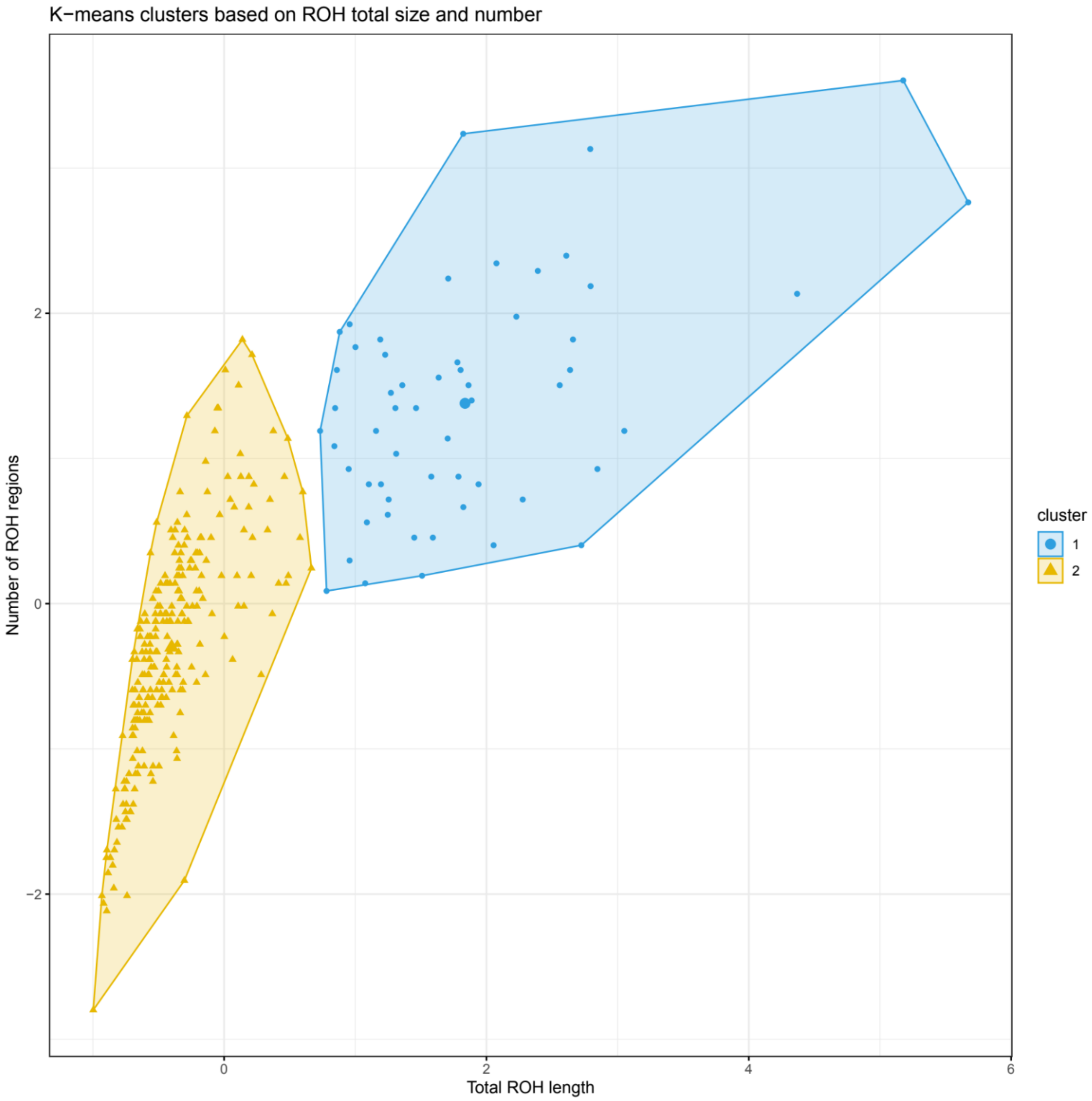

Supplementary Figure 12: Homozygosity levels in HLA alleles and autosomes in high-ROH and low-ROH samples. HLA-B and HLA-C genes show a significant difference in the allele homozygosity between high-ROH and low-ROH subjects. All HLA genes showed higher homozygosity levels than the autosomal level in high- and low-ROH samples.

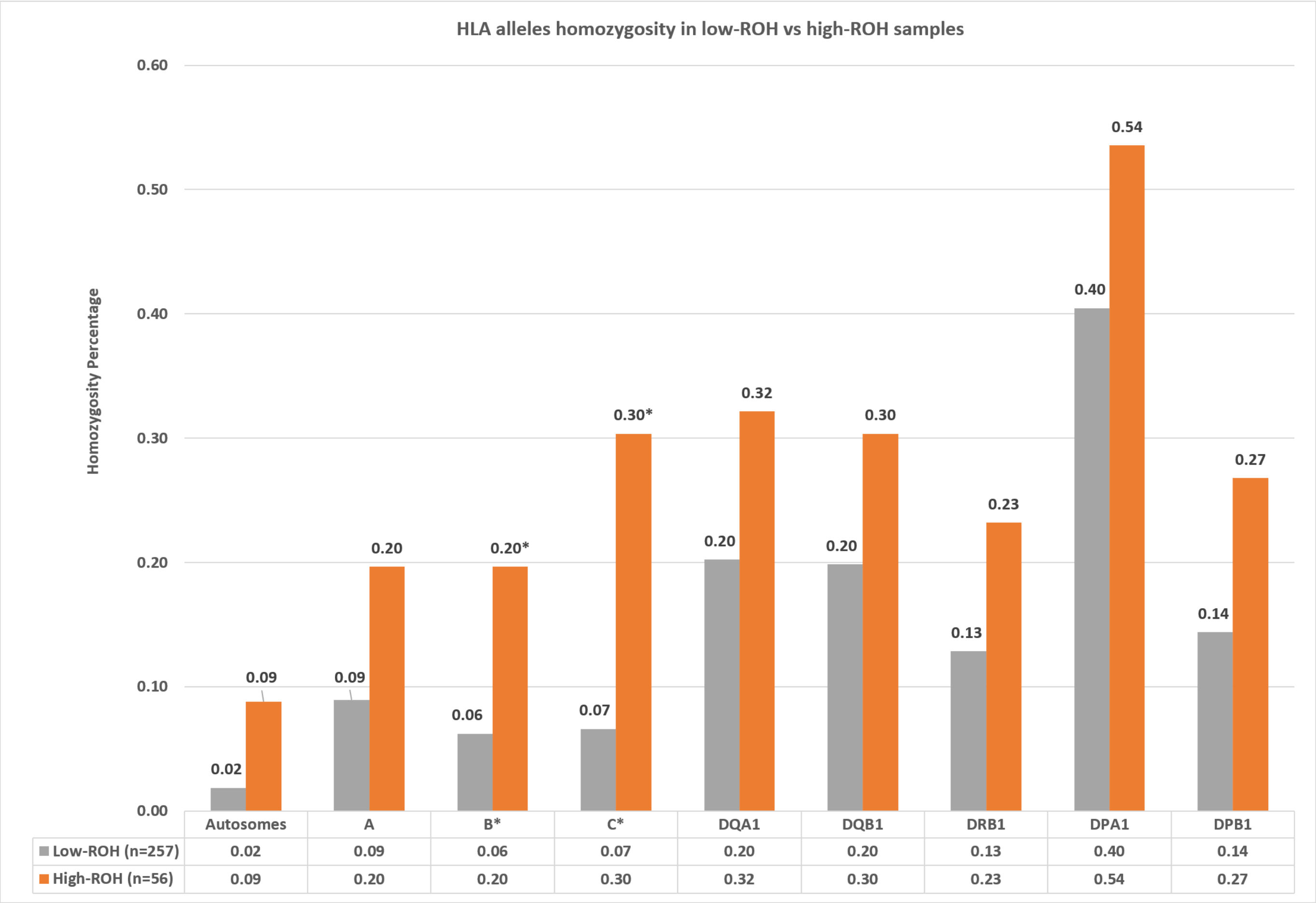

Supplement: Supplementary file 1 — Supplementary Information 1. [file 41598_2024_53986_MOESM1_ESM.pdf]
